# Low circulating levels of miR-17 and miR-126-3p are associated with increased mortality risk in geriatric hospitalized patients affected by cardiovascular multimorbidity

**DOI:** 10.1007/s11357-023-01010-1

**Published:** 2023-11-27

**Authors:** Francesca Marchegiani, Rina Recchioni, Mirko Di Rosa, Francesco Piacenza, Fiorella Marcheselli, Anna Rita Bonfigli, Roberta Galeazzi, Giulia Matacchione, Maurizio Cardelli, Antonio Domenico Procopio, Andrea Corsonello, Antonio Cherubini, Roberto Antonicelli, Giovanni Lombardi, Fabrizia Lattanzio, Fabiola Olivieri

**Affiliations:** 1 Clinic of Laboratory and Precision Medicine, IRCCS INRCA, 60121 Ancona, Italy; 2 Unit of Geriatric Pharmacoepidemiology and Biostatistics, IRCCS INRCA, 60124 Ancona, Italy; 3 Advanced Technology Center for Aging Research, IRCCS INRCA, 60121 Ancona, Italy; 4 Scientific Direction, IRCCS INRCA, Ancona, Italy; 5 Department of Clinical and Molecular Sciences, Università Politecnica Delle Marche, 60126 Ancona, Italy; 6 Unit of Geriatric Medicine, IRCCS INRCA, 87100 Cosenza, Italy; 7 Department of Pharmacy, Health and Nutritional Sciences, University of Calabria, 87036 Rende, Italy; 8 Geriatria, Accettazione Geriatrica e Centro Di Ricerca Per L’invecchiamento, IRCCS INRCA, 60127 Ancona, Italy; 9 Cardiology Unit, IRCCS INRCA, 60129 Ancona, Italy; 10 Laboratory of Experimental Biochemistry and Molecular Biology, IRCCS Istituto Ortopedico Galeazzi, Milan, Italy; 11 Department of Athletics, Strength and Conditioning, Poznań University of Physical Education, Poznań, Poland

**Keywords:** Geriatric patients, Multimorbidity, MicroRNAs, Mortality, CVD

## Abstract

MultiMorbidity (MM), defined as the co-occurrence of two or more chronic conditions, is associated with poorer health outcomes, such as recurrent hospital readmission and mortality. As a group of conditions, cardiovascular disease (CVD) exemplifies several challenges of MM, and the identification of prognostic minimally invasive biomarkers to stratify mortality risk in patients affected by cardiovascular MM is a huge challenge. Circulating miRNAs associated to inflammaging and endothelial dysfunction, such as miR-17, miR-21-5p, and miR-126-3p, are expected to have prognostic relevance. We analyzed a composite profile of circulating biomarkers, including miR-17, miR-21-5p, and miR-126-3p, and routine laboratory biomarkers in a sample of 246 hospitalized geriatric patients selected for cardiovascular MM from the Report-AGE INRCA database and BioGER INRCA biobank, to evaluate the association with all-cause mortality during 31 days and 12 and 24 months follow-up. Circulating levels of miR-17, miR-126-3p, and some blood parameters, including neutrophil to lymphocyte ratio (NLR) and eGFR, were significantly associated with mortality in these patients. Overall, our results suggest that in a cohort of geriatric hospitalized patients affected by cardiovascular MM, lower circulating miR-17 and miR-126-3p levels could contribute to identify patients at higher risk of short- and medium-term mortality.

**Supplementary Information:**

The online version contains supplementary material available at 10.1007/s11357-023-01010-1.

## Introduction

The rapid aging of the world’s population is accompanied by a substantial increase in the prevalence of multiple chronic diseases [1, 2]. The coexistance of two or more chronic diseases in the same individual has been defined as MultiMorbidity (MM) and complex multimorbidity (CMM) by the World Health Organization (WHO) [3]. Further, MM can coexist with frailty in older patients, and MM and frailty can interact to increase the risk of adverse outcomes [4, 5]. MM burdens individuals and health-care systems, increasing the likelihood of hospital admission, length of stay and readmission, healthcare costs, polypharmacy, and mortality, reducing the quality of life and independency [6, 7].

Besides, the pathophysiology of MM suffers of a knowledge gap, as well as the definition of its severity, and the association with related outcomes in different cohorts, differing in terms of age, gender, and pathological conditions [8]. Notably, cardiovascular and neuropsychiatric diseases represent the two major determinants of years of life spent with disability, especially in the setting of the older frail patient [4, 9]. In this context, the identification of prognostic, minimally invasive biomarkers allowing a stratification of mortality risk in patients affected by MM, especially cardiovascular MM, is a huge challenge.

Among the potential biomarkers, microRNAs (miRNAs), the shortest non-coding RNA species modulating gene expression, appear as promising biomarkers for monitoring the aging process and age-related disease development and progression [10, 11].

Several circulating miRNAs (c-miRNAs) have been deeply implicated in the development and progression of the most common age-related diseases, sharing inflammaging and endothelial dysfunction as common pathological mechanisms.

Specific miRNAs, such as miR-21-5p, miR-126-3p, and miR-17, were previously associated with age-related diseases, targeting molecular pathways underpinning the aging process in different tissues. Following the geroscience hypothesis, suggesting common pathways involved in aging and age-related diseases, these miRNAs were also proposed as molecular targets aimed at delaying the aging process as well as the onset of the most common age-related diseases [12, 13].

miR-21-5p and miR-126-3p, are recognized as “inflammamiRs”, playing a key role in inflammatory gene expression modulation, and their deregulation has been reported in several cancers (i.e., breast, lung, prostate cancers, and osteosarcoma) and the associated bone metastasis, with miR-21-5p being oncogenic and miR-126-3p being oncosuppressors [14], and in a wide variety of age-related diseases [15–17], as in osteoarthritis and osteoporosis [18]. In addition, miR-126-3p is also defined as an “angiomir” since it is involved in the modulation of vascular regeneration, and it has been extensively studied in patients affected by type 2 diabetes mellitus (T2DM) and CVD [19–22]. Finally, miR-126-5p has been recently identified as a key marker of muscle mass in post-menopausal osteoporotic women.

Also, miR-17, a microRNA belonging to the miR-17-92 cluster, has been extensively studied for its role in cancer [14, 23, 24], but it has been recognized to play important roles also in CVD, including heart failure (HF) [25–28] and in osteoporosis [29].

In summary, we select these three miRNAs, namely, miR-17, miR-21-5p, and miR-126-3p, as they cover relevant roles in several pathological conditions associated with aging and, above all, associated with aging outcomes, but no data are available on them as mortality risk predictors.

Therefore, in the present study, their plasma levels were analyzed in 246 selected patients from the Report-AGE INRCA project, representing hospitalized geriatric patients with cardiovascular MM, with the aim to estimate the association with all-cause mortality during different follow-up periods (31 days and 12–24 months). Understanding the heterogeneity of the aging trajectories, predicting or tracking MM development, and ultimately stratifying older patients based on the risk of death hold the promise of applying precision medicine to geriatric patients.

## Patients and methods

### Study population

A cohort of 246 patients was selected from the Report-AGE project, based on the following criteria: evidence for cardiovascular MM, availability of plasma samples, routine biomarker measurements (hemoglobin, WBC, RBC, platelets, lymphocytes, neutrophils, monocytes, basophils and eosinophils counts, BUN, creatinine, sodium, potassium, and GFR), and complete clinical information. The Report-AGE project is a large-scale observational study about the health conditions of older patients (> 65 years) hospitalized at INRCA Research Institute [30] (Trial Registration no. NCT01397682). For this study, patients admitted between June 16, 2012, and November 3, 2017, were included. Blood samples were collected in EDTA tubes (Becton, Dickinson and Company, New Jersey, USA) within the first 24 h following the hospital admission and stored in BioGer INRCA biobank until the analysis of miRNAs expression. Blood samples were centrifuged, within 2 h from the collection, at 1800 RPM for 10 min to separate plasma. Plasma was then aliquoted and frozen at − 80 °C. Mortality was assessed at 31 days and 12 and 24 months of follow-up. Data on disease history of all patients, recruited for this study, were obtained from the medical records. All diagnoses are coded in accordance with the International Classification of Diseases, 9th revision (http://www.icd9data.com/). Patients affected by only one chronic disease have been excluded, and in order to limit any statistical noise and the risk of spurious results, diseases with a prevalence < 2% were excluded. The inclusion criterion of cardiovascular MM was verified based on the presence of at least one diagnosis (at admission and/or earlier) with one or more of the following ICD-9: 402* and 404* (“hypertensive disease category”), 410*–414* (“ischemic heart disease category”), and 427* and 428* (“other forms of heart disease”). The presence of comorbidities was established by the ICD-9, too; codes for each comorbidity were reported in supplementary table 1. To identify frail older patients, the Hospital Frailty Risk Score (HFRS) was applied (Gilbert et al., 2018). HFRS is categorized as low (< 5 points), intermediate (5–15 points), or high (> 15 points).

### MicroRNAs analysis

Total RNA was isolated from a plasma sample (100 µl), stored in BioGer biobank INRCA, Ancona, using a total RNA purification kit by Norgen Biotek Corporation, according to the manufacturer’s specific recommendations. The levels of the hsa-miR-17, hsa-miR-21-5p, and hsa-miR-126-3p were determined using TaqMan MicroRNA assays (ThermoFisher Scientific). MicroRNAs were reverse-transcribed using a TaqMan microRNA RT kit (catalog number 4366596) and the associated miRNA-specific stem-loop primers. Synthetic cel‐miR‐39-3p was spiked‐in before RNA isolation for normalization in subsequent qRT‐PCR. Expression levels of the measured miRNAs were normalized against cel-miR-39-3p. The 2^−ΔCt^ method was applied to obtain the miRNA relative expression level for each patient (ΔCt = Ct value of the reference miRNA subtracted from the Ct value of the target miRNA).

### Routine laboratory biomarkers

Hemoglobin, WBC, RBC, platelets, lymphocytes, neutrophils, monocytes, basophils, and eosinophils counts were performed by standard procedures. BUN, creatinine, sodium, and potassium were measured by routine laboratory methods. GFR was estimated according to Chronic Kidney Disease Epidemiology Collaboration (CKD-EPI) equation [31].

### Diana miRPath.v4 tool

For the identification of miR-17 and miR-126-3p-associated pathways and targets, we took advantage of the online miRNA analysis platform DIANA-miRPath (v4.0, http://www.microrna.gr/miRPathv4), that harness predicted or experimentally supported miRNA interactions towards the exploration of combined miRNA effects [32]. Experimentally verified interactions were retrieved from the reference resources DIANA-TarBase v8. MiRNA-centric analysis was performed by using two different merging methods: the pathway union and the genes intersection. KEGG pathways were selected, and the method analysis set the significance at *p* < 0.05 and the FDR correction.

### Statistical analyses

Continuous variables’ distribution was assessed via the Kolmogorov–Smirnov test. Categorical variables were presented as proportions, continuous variables as means (standard deviation) for normal distributed variables, and median (interquartile range) for non-normal distributed variables. Differences in clinical characteristics according to different mortality rates (at 31 days FU, 12 months FU, and 24 months FU) were compared by the chi‐squared test (*χ*^2^) for categorical variables; for continuous variables, Student’s *t*-test or Wilcoxon rank-sum test were used according to the variable’s distribution. Cox proportional hazards regression analysis was used to estimate hazard ratios (HR) and 95% confidence intervals (95% CI) of the statistically significant variables in bivariate analysis with respect to the mortality rate occurrence. Kaplan Meier Survival analysis and the log‐rank test were used to estimate and compare the occurrence of death within the three times of follow-up period. All the microRNAs plasma levels were also dichotomized based on the upper quartile as the cut-off point. Multivariable Cox proportional hazards analysis for dichotomized microRNAs (adjusted for age, gender, and confounders statistically significant for all the endpoints avoiding overlapping and potential multicollinearity) were also estimated. All tests were two-sided, and significance was set at *p* < 0.05. Statistical analyses were performed using IBM SPSS version 25.0 and STATA version 15.1 Statistical Software Package for Windows (Stata Corp, College Station, TX, USA).

## Results

### Patients characteristics association with survival outcome

The clinical characteristics of the 246 selected patients, divided based on survival status and grouped based on the follow-up duration (31 days, 12 months, and 24 months FU), are reported in Table 1. At baseline, study participants’ median age (IQR) was 86 (83–90) years, and females were 56.9%. As expected, older patients were significantly more represented among deceased patients for all the three follow-up periods considered (*p* < 0.05 for 31 days, 12 months and 24 months FU, Table 1). Due to the selection strategy, the comorbidity with the highest prevalence was CHF (82.9%), followed by hypertension (HTN), CKD, cardiac arrhythmias, COPD, diabetes/dyslipidemia, CAD/PAD, acute diseases of the digestive system, endocrine/nutritional and metabolic diseases, pneumonia, degenerative diseases of the CNS, acute diseases of the urinary system, chronic diseases of the digestive system, cancer, bone and muscle diseases, unspecified pleural effusion, and, lastly, stroke (2.8%). The impact of these diseases on death risk at the defined time-points is different: a statistically significant difference between survived and deceased patients was found for stroke considering in-hospital mortality (31 days FU), for HTN considering 12 months FU, and for degenerative diseases of the CNS considering 24 months FU (*p* < 0.05, Table 1). In deceased patients, the rates for stroke and degenerative diseases of the CNS were higher than in survived patients (10.3% vs. 1.8%, 19.1% vs. 10%, and 38.2% vs. 25.5%, respectively, Table 1). Notably, the proportion of HTN was significantly higher in survived patients than in deceased patients (85.4% vs. 71.6% for HTN). These paradoxical results should be contextualized within the therapy regimens and the overall complex clinical evaluation of the patients. Overall, during the 24-month follow-up, 136 (55.3%) deaths occurred with a mean of time to death of 14 months; this is not a surprising result, considering the very old age of this cohort. Interestingly, HFRS was not a determinant for the 31-day risk of death, but it becomes statistically significant for the 12- and 24-month FU. In particular, the “intermediate risk category” appears to have the highest risk of death. About the diagnosis at entrance, CHF has the highest prevalence (21.3%) followed by pneumonia (data not shown). No patients with diabetes/dyslipidemia were hospitalized for these conditions.

**Table 1:** Clinical characteristics of the 246 selected patients, grouped based on the follow-up times (31 days, 12 months, and 24 months FU)

		31 days FU			12 months FU			24 months FU		
	Total *n* = 246	Survived *n* = 217	Deceased *n* = 29	*p*	Survived *n* = 137	Deceased *n *=109	*p*	Survived *n* = 110	Deceased *n *= 136	*p*
Female gender, *n* (%)	140 (56.9%)	124 (57.1%)	16 (55.2%)	0.841	77 (56.2%)	63 (57.8%)	0.802	66 (60%)	74 (54.4%)	0.379
Age, median (IQR)	86 (83–90)	86 (83–90)	89 (87–91)	**0.004**	85 (82–89)	88 (84–92)	** < 0.001**	85 (82–89)	88 (84–91)	**0.002**
Comorbidities										
CHF, *n* (%)	204 (82.9%)	183 (84.3%)	21 (72.4%)	0.109	119 (86.9%)	85 (78%)	0.066	93 (84.5%)	111 (81.6%)	0.544
HTN, *n* (%)	195 (79.3%)	176 (81.1%)	19 (65.5%)	0.052	117 (85.4%)	78 (71.6%)	**0.008**	93 (84.5%)	102 (75%)	0.066
Chronic kidney disease, *n* (%)	111 (45.1%)	100 (46.1%)	11 (37.9%)	0.407	58 (42.3%)	53 (48.6%)	0.325	45 (40.9%)	66 (48.5%)	0.232
Cardiac arrhythmias, *n* (%)	76 (30.9%)	70 (32.3%)	6 (20.7%)	0.205	45 (32.8%)	31 (28.4%)	0.457	34 (30.9%)	42 (30.9%)	0.996
COPD, *n* (%)	70 (28.5%)	60 (27.6%)	10 (34.5%)	0.444	33 (24.1%)	37 (33.9%)	0.089	25 (22.7%)	45 (33.1%)	0.073
Diabetes/dyslipidemia, *n* (%)	61 (24.8%)	51 (23.5%)	10 (34.5%)	0.198	34 (24.8%)	27 (24.8%)	0.993	28 (25.5%)	33 (24.3%)	0.830
CAD/PAD, *n* (%)	54 (22%)	46 (21.2%)	8 (27.6%)	0.435	32 (23.4%)	22 (20.2%)	0.550	28 (25.5%)	26 (19.1%)	0.233
Acute diseases of the digestive system, *n* (%)	48 (19.5%)	43 (19.8%)	5 (17.2%)	0.742	25 (18.2%)	23 (21.1%)	0.575	21 (19.1%)	27 (19.9%)	0.881
Endocrine, nutritional and metabolic diseases, *n* (%)	41 (16.7%)	35 (16.1%)	6 (20.7%)	0.536	19 (13.9%)	22 (20.2%)	0.187	15 (13.6%)	26 (19.1%)	0.251
Pneumonia, *n* (%)	39 (15.9%)	33 (15.2%)	6 (20.7%)	0.448	22 (16.1%)	17 (15.6%)	0.921	18 (16.4%)	21 (15.4%)	0.844
Degenerative diseases of the CNS, *n* (%)	37 (15%)	33 (15.2%)	4 (13.8%)	0.841	18 (13.1%)	19 (17.4%)	0.349	11 (10%)	26 (19.1%)	**0.047**
Acute diseases of the urinary system, *n* (%)	36 (14.6%)	33 (15.2%)	3 (10.3%)	0.487	19 (13.9%)	17 (15.6%)	0.703	15 (13.6%)	21 (15.4%)	0.690
Chronic diseases of the digestive system, *n* (%)	31 (12.6%)	29 (13.4%)	2 (6.9%)	0.324	18 (13.1%)	13 (11.9%)	0.776	16 (14.5%)	15 (11%)	0.409
Cancer, *n* (%)	28 (11.4%)	23 (10.6%)	5 (17.2%)	0.290	14 (10.2%)	14 (12.8%)	0.520	9 (8.2%)	19 (14%)	0.155
Bone and muscle diseases, *n* (%)	25 (10.2%)	25 (11.5%)	0 (0%)	0.054	16 (11.7%)	9 (8.3%)	0.378	10 (9.1%)	15 (11%)	0.617
Unspecified pleural effusion, *n* (%)	21 (8.5%)	19 (8.8%)	2 (6.9%)	0.736	10 (7.3%)	11 (10.1%)	0.436	7 (6.4%)	14 (10.3%)	0.273
Stroke, *n* (%)	7 (2.8%)	4 (1.8%)	3 (10.3%)	**0.010**	3 (2.2%)	4 (3.7%)	0.488	3 (2.7%)	4 (2.9%)	0.920
Hospital Frailty Risk Score (HFRS), n (%)				0.906			**0.036**			**0.004**
Low risk	62 (25.2%)	55 (25.3%)	7 (24.1%)		44 (32.1%)	18 (16.5%)		40 (36.4%)	22 (16.2%)	
Intermediate risk	169 (68.7%)	148 (68.2%)	21(72.4%)		84 (61.3%)	85 (78.0%)		64 (58.2%)	105 (77.2%)	
High risk	13 (5.3%)	12 (5.5%)	1(3.4%)		8 (5.8%)	5 (5.6%)		5 (4.5%)	8 (5.9%)	
miRNAs										
miR–17, median (IQR)	2.20 (1.06–4.58)	2.51 (1.16–4.61)	1.46 (0.72–2.19)	**0.009**	2.52 (1.16–5.15)	2.10 (0.96–3.78)	0.122	2.61 (1.20–5.82)	2.03 (0.97–3.90)	0.062
miR-17 ≥ 4.59, *n* (%)	61 (24.8%)	57 (26.3%)	4 (13.8%)	0.144	40 (29.2%)	21 (19.3%)	0.073	34 (30.9%)	27 (19.9%)	**0.046**
miR-126-3p, median (IQR)	3.76 (1.88–6.64)	4.02 (2.07–6.81)	2.08 (1.63–4.46)	**0.025**	4.03 (2.05–8.29)	2.87 (1.88–5.80)	0.156	4.15 (2.18–8.89)	3.13 (1.77–5.77)	0.052
miR-126-3p ≥ 6.66, *n* (%)	61 (24.8%)	57 (26.3%)	4 (13.8%)	0.144	42 (30.2%)	19 (17.8%)	**0.017**	37 (33.6%)	24 (17.6%)	**0.004**
miR-21-5p, median (IQR)	0.75 (0.43–1.31)	0.75 (0.43–1.32)	0.82 (0.48–1.13)	0.935	0.76 (0.40–1.31)	0.73 (0.45–1.30)	0.665	0.74 (0.42–1.31)	0.79 (0.43–1.31)	0.637
miR-21-5p ≥ 1.32, *n* (%)	61 (24.8%)	55 (25.3%)	6 (20.7%)	0.586	34 (24.8%)	27 (24.8%)	0.993	27 (24.5%)	34 (25%)	0.935
Clinical biochemistry										
WBC, median (IQR)	8.03 (6.23–10.63)	7.94 (6.15–10.25)	10.49 (6.95–13.93)	**0.010**	7.74 (5.98–10.07)	8.53 (6.53–11.64)	**0.015**	7.72 (6.06–10.03)	8.44 (6.31–11.30)	0.052
RBC, mean ± sd	3.85 ± 0.66	3.86 ± 0.63	3.77 ± 0.81	0.482	3.93 ± 0.62	3.74 ± 0.68	**0.023**	3.93 ± 0.60	3.78 ± 0.69	0.069
HGB, median (IQR)	10.9 (9.7–12.3)	11.0 (9.6–12.4)	10.3 (9.7–11.7)	0.372	11.4 (9.7–12.7)	10.6 (9.7–11.6)	**0.018**	11.5 (9.7–12.8)	10.6 (9.6–11.7)	**0.029**
HCT, median (IQR)	33.6 (29.3–37.5)	33.8 (29.5–37.6)	31.6 (29.3–35.7)	0.312	34.6 (29.7–38.1)	32.4 (28.9–36.3)	**0.024**	34.6 (29.7–38.4)	32.7 (29.0–36.6)	**0.044**
PLT, median (IQR)	204 (162–271)	206 (162–274)	204 (170–222)	0.959	212 (163–259)	201 (160–271)	0.575	205 (160–259)	204 (162–271)	0.899
Neutrophils %, median (IQR)	76.0 (68.1–83.4)	75.2 (66.0–82.3)	82.1 (75.3–89.0)	** < 0.001**	73.8 (64.4–81.2)	78.8 (72.3–86.4)	** < 0.001**	73.1 (63.9–81.8)	78.5 (71.8–84.3)	**0.001**
Lymphocytes %, median (IQR)	14.3 (10.0–21.1)	14.8 (10.3–22.4)	10.4 (6.6–13.1)	** < 0.001**	16.2 (11.3–24.2)	13.1 (7.8–17.3)	** < 0.001**	16.4 (11.3–24.4)	13.1 (8.2–18.2)	**0.001**
Monocytes %, median (IQR)	7.0 (5.0–9.0)	7.3 (5.1–9.0)	5.4 (4.0–7.5)	**0.001**	7.9 (6.0–9.4)	6.3 (4.2–8.1)	** < 0.001**	7.7 (5.9–9.4)	6.7 (4.6–8.4)	**0.010**
Eosinophils %, median (IQR)	0.6 (0–2)	0.6 (0.1–2.2)	0.1 (0.0–1.1)	0.088	0.8 (0.1–2.3)	0.2 (0.0–1.6)	**0.004**	0.9 (0.1–2.3)	0.2 (0.0–1.6)	**0.003**
Basophils %, median (IQR)	0.2 (0.1–0.4)	0.2 (0.1–0.4)	0.2 (0.1–0.3)	0.969	0.2 (0.1–0.4)	0.2 (0.1–0.3)	0.193	0.2 (0.1–0.4)	0.2 (0.1–0.3)	0.121
NLR, median (IQR)	5.28 (3.24–8.25)	5.00 (2.97–7.84)	7.60 (5.48–13.14)	** < 0.001**	4.69 (2.69–7.19)	6.00 (4.19–11.15)	** < 0.001**	4.56 (2.56–7.19)	5.83 (3.92–10.33)	**0.001**
BUN, median (IQR)	64 (42–98)	59 (40–87)	101 (85–153)	**0.001**	53 (37–77)	81 (51–118)	** < 0.001**	54 (37–77)	74 (46–111)	** < 0.001**
Creatinine, median (IQR)	1.2 (0.9–1.6)	1.1 (0.9–1.6)	1.6 (1.3–2.6)	**0.001**	1.1 (0.9–1.5)	1.4 (1.0–2.1)	** < 0.001**	1.1 (0.9–1.5)	1.3 (0.9–1.9)	**0.027**
eGFR, median (IQR)	45 (31–67)	48 (33–69)	33 (20–42)	**0.002**	54 (36–70)	39 (21–57)	** < 0.001**	49 (34–69)	42 (27–63)	**0.019**
Sodium, median (IQR)	140 (137–142)	139 (137–142)	141 (137–143)	0.521	140 (137–142)	140 (136–143)	0.747	140 (137–142)	139 (136–142)	0.485
Potassium, median (IQR)	4.1 (3.8–4.6)	4.1 (3.8–4.6)	4.3 (3.9–4.7)	0.879	4.1 (3.8–4.6)	4.1 (3.8–4.6)	0.468	4.0 (3.7–4.6)	4.2 (3.9–4.6)	0.055

*CHF*, chronic heart failure; *HTN*, hypertension; *COPD*, chronic obstructive pulmonary disease; *CAD/PAD*, coronary artery disease/ peripheral artery disease; *HFRS*, Hospital Frailty Risk Score; *WBC*, white blood cell; *RBC*, red blood cell; *HGB*, haemoglobin; *HCT*, haematocrit; *PLT*, platelets; *NLR*, neutrophil-to-lymphocyte ratio; *BUN*, blood urea nitrogen; *eGFR*, estimated glomerular filtration rate; *IQR*, interquartile range. MiRNAs are reported as relative expression. In bold significant variables

### Patients characteristics and circulating biomarker: associations with survival outcome

To estimate the association of miR-17, miR-21-5p, and miR-126-3p with mortality over the different FU periods, we tested miRNAs expression levels both as continuous and dichotomous variables, the upper quartile vs. all the other values. The results of these univariate analyses are reported in Table 1. miR-17 and miR-126-3p resulted significantly associated with mortality. When miR-17 expression levels were analyzed as continuous values, a statistically significant association was observed with mortality at 31 days FU (miR-17 median value of 2.51 for survived patients vs. miR-17 median value of 1.46 for deceased patients, *p* = 0.009). When miR-17 expression levels were analyzed as dichotomized values, a significant association with mortality at 24 months FU was found (30.9% of survived patients vs 19.9% of deceased with miR-17 ≥ 4.59, *p* = 0.046). For miR-126-3p, a statistically significant association with mortality at 31 days FU was observed for continuous values (miR-126-3p median value of 4.02 for survived patients vs miR-126-3p median value of 2.08 for deceased patients, *p* = 0.025). Interestingly, when miR-126-3p expression levels were analyzed as dichotomized values, miR-126-3p levels ≥ 6.66 were statistically more represented among survived patients both at 12- and 24-months FU (*p* = 0.017 and *p* = 0.004, respectively), suggesting a protective effect of this miRNA on mortality.

Notably, on the entire sample, a strong correlation between miR-17 and miR-126-3p expression levels was found (Pearson’s correlation 0.916, *p* < 0.001, data not shown).

On the contrary, no correlation between miR-21-5p and mortality at any FU periods was found (*p* not significant, Table 1).

Among routine blood parameters, a significant association with 31-day mortality was observed for the following biomarkers: WBC, neutrophils %, lymphocytes %, monocytes %, neutrophil–lymphocyte ratio (NLR), BUN, creatinine, and eGFR. These parameters resulted significantly higher in deceased patients compared to survived patients, except for lymphocytes, monocytes, and eGFR. Biochemical parameters significantly associated with 12-month mortality were WBC, RBC, HGB, HCT, neutrophils %, lymphocytes %, monocytes %, eosinophils %, NLR, BUN, creatinine, and eGFR. Finally, parameters associated with 24-month mortality were the same as observed for 12 months mortality except for WBC and RBC that, in this case, did not reach the statistical significance. Among the hematological parameters, neither platelets nor basophils were associated with mortality at any FU time-point. Similarly, among the biochemical parameters, no association was observed for sodium and potassium.

### Univariate and multivariate Cox proportional hazards regression analysis of overall survival at different FU

**Fig. 1:**
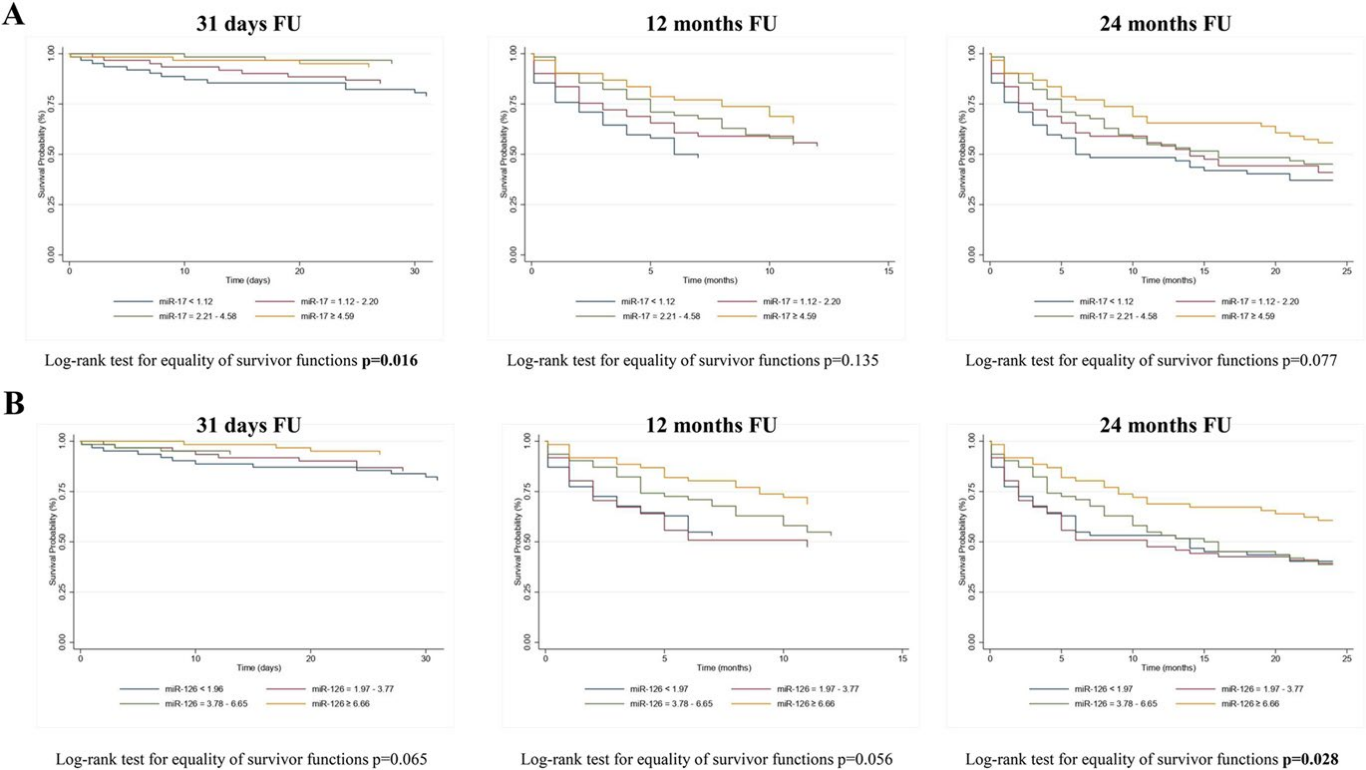
Kaplan–Meier curves for miRNAs quartiles. Kaplan–Meier survival function for patients according to (**A**) miR-17 quartiles plasma levels and (**B**) miR-126 quartiles plasma levels in respect to the three times FU (31 days, 1 year, and 2 years). MiRNAs are reported as relative expression

**Fig. 2:**
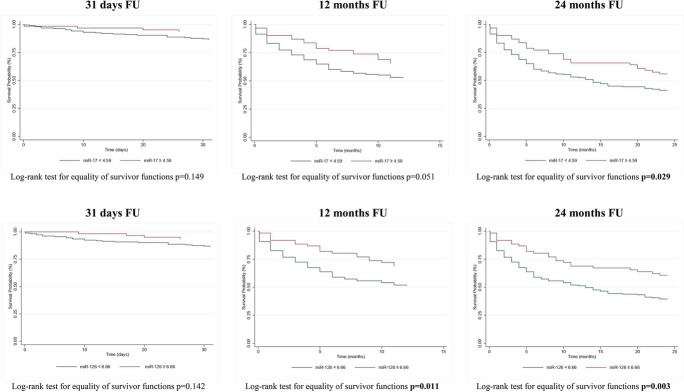
Kaplan–Meier curves for highest quartile of miRNA. Kaplan–Meier survival function for patients according to (**A**) miR-17 (upper quartile vs lowest quartile) and (**B**) miR-126 (upper quartile vs lowest quartile) plasma levels in respect to the three times FU (31 days, 1 year, and 2 years). MiRNAs are reported as relative expression

To estimate the association between miR-17 and miR-126-3p and the survival rate, we analyzed miRNAs expression levels in terms of quartiles and of dichotomous variable (highest quartile of miRNAs expression levels vs. all other quartiles), over the three time-points. Kaplan–Meier curves for miRNAs quartiles and for the highest quartile of miRNAs vs. the others are reported in Figs. 1 and 2, respectively. The quartiles-based analysis in Fig. 1 showed that patients with the lowest levels of miR-17 had a significantly higher risk mortality at 31 days (*p* = 0.016); conversely, the higher risk of mortality at 24-month FU was observed for patients with the lowest levels of miR-126-3p (*p* = 0.028). When we considered the highest quartile of miR-17 and miR-126-3p, the log-rank test showed that patients with the highest levels of miR-17 (higher than 4.59) and miR-126-3p (higher than 6.66) had a better prognosis at 24 months FU (*p* = 0.029 and *p* = 0.003, respectively). Moreover, the highest levels of miR-126-3p were positively associated with a good prognosis also at 12 months FU.

To evaluate the prognostic accuracy of all the variables resulted statistically significant in Table 1, the crude hazard ratio and CI 95% were evaluated through a logistic regression (see Table 2). Neither miR-17 nor miR-126-3p was found predictive of death rate, at any of the time-point. Concerning comorbidities, only HTN and stroke have been confirmed to be predictors of death. In particular, stroke reached statistical significance with a HR of 4.73 (1.43–15.64) for 31-day mortality. Surprisingly, patients with HTN would appear to be protected from the risk of death over all the FU considered (HR of 0.45 for 31-day FU, HR of 0.54 for 12-month FU, HR of 0.61 for 24-month FU). These results could be explained, at least partly, by the ongoing pharmacological treatments. Crude hazard ratio for HFRS confirmed the results reported in Table 1; patients belonging to the intermediate risk category have a higher risk of 12- and 24-month FU mortality (HR 1.88 (1.13–3.13) for 12-month FU and 2.06 (1.30–3.27) for 24-month FU, Table 2). Regarding biochemical parameters, crude HR confirmed the results already reported in Table 1. In particular, RBC, HGB, lymphocytes%, monocytes%, eosinophils%, and eGFR showed significantly crude HR < 1, suggesting that patients with low values of these biomarkers were at higher risk of death at the different time-points considered (Table 2). Conversely, WBC, HCT, neutrophils%, BUN, creatinine, and NLR showed significantly crude HR > 1, suggesting that patients with high values of these parameters were at higher risk of death at the different time-points considered (Table 2).

**Table 2:** Univariate Cox proportional hazards regression analysis of overall survival at different FU

	31 days FU	12 months FU	24 months FU
	HR (95%CI)	HR (95%CI)	HR (95%CI)
HTN	**0.45 (0.21–0.98)**	**0.54 (0.35–0.82)**	**0.61 (0.42–0.91)**
Degenerative diseases of the CNS	0.89 (0.31–2.55)	1.31 (0.80–2.15)	1.53 (0.99–2.35)
Stroke	**4.73 (1.43–15.64)**	1.83 (0.68–4.98)	1.41 (0.52–3.81)
HFRS, ref. low risk			
Intermediate risk	1.08 (0.46–2.55)	**1.88 (1.13–3.13)**	**2.06 (1.30–3.27)**
High risk	0.66 (0.08–5.34)	1.26 (0.47–3.40)	1.76 (0.78–3.96)
miR-17	0.99 (0.95–1.04)	1.00 (0.98–1.02)	0.99 (0.97–1.01)
miR-126-3p	0.94 (0.86–1.03)	1.00 (0.98–1.01)	0.99 (0.98–1.01)
WBC	1.01 (0.99–1.02)	1.01 (0.99–1.01)	**1.01 (1.00–1.01)**
RBC	0.80 (0.46–1.41)	**0.68 (0.50–0.92)**	**0.73 (0.56–0.96)**
HGB	0.92 (0.75–1.12)	**0.87 (0.78–0.97)**	**0.88 (0.80–0.97)**
HCT	0.96 (0.90–1.03)	**0.96 (0.92–0.99)**	**0.96 (0.93–0.99)**
Neutrophils %	**1.07 (1.03–1.12)**	**1.03 (1.02–1.05)**	**1.03 (1.01–1.04)**
Lymphocytes %	**0.90 (0.85–0.96)**	**0.96 (0.94–0.99)**	**0.97 (0.95–0.99)**
Monocytes %	**0.88 (0.78–0.99)**	**0.88 (0.83–0.94)**	**0.92 (0.87–0.97)**
Eosinophils %	**0.68 (0.47–0.99)**	0.92 (0.82–1.04)	0.93 (0.84–1.03)
BUN	**1.01 (1.01–1.01)**	**1.01 (1.01–1.01)**	**1.01 (1.00–1.01)**
Creatinine	**1.38 (1.12–1.69)**	**1.30 (1.14–1.48)**	**1.25 (1.10–1.43)**
eGFR	**0.96 (0.94–0.98)**	**0.98 (0.97–0.99)**	**0.99 (0.98–0.99)**
NLR	**1.07 (1.03–1.11)**	**1.06 (1.03–1.08)**	**1.05 (1.02–1.08)**

*HTN*, hypertension; *HFRS*, Hospital Frailty Risk Score; *WBC*, white blood cell; *RBC*, red blood cell; HGB, haemoglobin; *HCT*, haematocrit; *BUN*, blood urea nitrogen; *eGFR*, estimated glomerular filtration rate; *NLR*, neutrophil-to-lymphocyte ratio. MiRNAs are reported as relative expression. In bold significant variables

Since the purpose of this study was to investigate the role of miRNAs in predicting the risk of death within a group of older patients with cardiovascular MM, the results of the adjusted Cox regression analysis of survival are reported in Table 3. The adjusted HR for miR-17 ≥ 4.59 at 24 months FU was 0.64 (0.42–0.97), suggesting that high levels of miR-17 could be protective for the risk of death at 2 years (Table 3). Age, NLR, and eGFR were confirmed as predictors of mortality.

**Table 3:** Multivariate Cox proportional hazards regression analysis of overall survival at different FU

	31 days FU	12 months FU	24 months FU
	HR (95%CI)	HR (95%CI)	HR (95%CI)
miR-17 ≥ 4.59	0.56 (0.19–1.63)	0.65 (0.40–1.06)	**0.64 (0.42–0.97)**
Age	**1.07 (1.00–1.14)**	**1.05 (1.02–1.09)**	**1.05 (1.02–1.08)**
Female gender	1.57 (0.71–3.47)	1.25 (0.83–1.86)	1.37 (0.96–1.95)
NLR	1.04 (0.99–1.09)	**1.04 (1.01–1.07)**	**1.04 (1.01–1.07)**
eGFR	**0.97 (0.95–0.99)**	**0.99 (0.98–1.00)**	0.99 (0.98–1.00)
HTN	0.62 (0.27–1.40)	0.67 (0.43–1.04)	0.73 (0.48–1.10)
miR–126–3p ≥ 6.66	0.51 (0.17–1.46)	**0.56 (0.34–0.92)**	**0.54 (0.35–0.84)**
Age	1.06 (0.99–1.13)	**1.05 (1.01–1.08)**	**1.05 (1.01–1.08)**
Female gender	1.56 (0.71–3.45)	1.24 (0.83–1.86)	1.37 (0.96–1.95)
NLR	1.04 (0.99–1.09)	**1.04 (1.01–1.07)**	**1.04 (1.01–1.07)**
eGFR	**0.97 (0.94–0.99)**	**0.99 (0.98–1.00)**	0.99 (0.98–1.00)
HTN	0.62 (0.28–1.40)	0.67 (0.43–1.05)	0.74 (0.49–1.11)

*NLR*, neutrophil-to-lymphocyte ratio; *eGFR*, estimated glomerular filtration rate; *HTN*, hypertension. MiRNAs are reported as relative expression. In bold significant variables

The adjusted HR for miR-126-3p ≥ 6.66 was 0.56 (0.34–0.92) at 12 months FU and 0.54 (0.35–0.84) at 24 months FU, suggesting that high miR-126 levels are protective for risk of death at 1–2 years from hospitalization (Table 3).

Notably, in both cases, female gender and hypertension were not associated with the risk of death.

### miR-17 and miR-126-3p pathway analysis

To improve the functional characterization of selected miRNAs, we performed the pathway analysis through the platform DIANA-miRPath. The Pathway Union study revealed all the pathways in which miR-126-3p and miR-17 are involved, as well as the number of targeted mRNAs. We selected 21 pathways containing mRNAs targeted by miR-126-3p and miR-17. Some of these pathways are associated with human diseases, such as cancer, metabolic diseases, and atherosclerosis, and mechanisms involved in the aging process, such as autophagy, cellular senescence, and longevity regulating pathways (Table 4).

**Table 4:** Pathway union analysis of miR-126-3p and miR-17 by miRPath.v4

Term name	Target genes (*n*)	Merged *P*-value	Merged FDR
FoxO signaling pathway	42,8	4.01506E-13	4.54E-11
Autophagy—animal	42,7	1.85298E-11	6.98E-10
Chronic myeloid leukemia	28,6	1.38679E-11	6.98E-10
Endocrine resistance	35,7	4.53899E-11	1.28E-09
Neurotrophin signaling pathway	34,8	9.74515E-11	1.84E-09
Shigellosis	62,9	9.01213E-11	1.84E-09
Pathways in cancer	102,13	1.49729E-09	1.97E-08
Prostate cancer	28,6	1.38697E-08	1.12E-07
Breast cancer	39,7	2.11124E-08	1.59E-07
Small cell lung cancer	25,7	3.40726E-08	2.41E-07
Cellular senescence	47,8	5.69919E-08	3.31E-07
Non-small cell lung cancer	22,6	6.54311E-08	3.52E-07
Gastric cancer	34,8	2.40543E-07	1.07E-06
Longevity regulating pathway	24,7	2.60082E-07	1.09E-06
AMPK signaling pathway	31,6	3.88654E-07	1.42E-06
Longevity regulating pathway—multiple species	17,7	5.39146E-07	1.85E-06
PI3K-Akt signaling pathway	64,11	1.12082E-06	3.52E-06
EGFR tyrosine kinase inhibitor resistance	21,5	1.99571E-06	5.5E-06
Fluid shear stress and atherosclerosis	33,6	1.93349E-06	5.5E-06
Endometrial cancer	18,4	2.10061E-06	5.65E-06
Growth hormone synthesis, secretion and action	28,6	4.5499E-06	1.2E-05

The Target Union analysis highlighted 7 mRNAs targets of both miR-126-3p and miR-17. Table 5 displays the gene target corresponding IDs, as obtained by the DIANA miRpath.v4 analysis.

**Table 5:** Target union analysis of miR-126-3p/miR-17 by miRPath.v4

Target gene name	Target gene IDs
DICER1	ENSG00000100697
E2F1	ENSG00000101412
E2F3	ENSG00000112242
MCL1	ENSG00000143384
IGF2BP1	ENSG00000159217
CRK	ENSG00000167193
BCL2	ENSG00000171791

## Discussion

More than 50% of the older patients suffer from more than two diseases at the same time, a condition defined MM. MM is associated with an increased risk of adverse outcomes, such as disability, institutionalization, loss of self-sufficiency, rehospitalizations, greater use of healthcare resources, and, ultimately, death [33–35]. The identification of biomarkers potentially predicting the mortality risk, and/or the risk of rehospitalization that represents itself a risk factor for mortality in the older population, becomes an increasingly important need. To address this issue, we have selected from the Report-AGE INRCA study, a group of geriatric patients with cardiovascular MM, to investigate the potential role of three miRNAs, known to be associated with several pathological conditions of the elderly, i.e., miR-17, miR-21-5p, and miR-126-3p, as predictive mortality biomarkers at different times of FU (31 days, 12 months, and 24 months).

As expected, age was the most reliable predictor of death. Notably, despite the large number of comorbidities, only 3 diseases, i.e., HTN, stroke, and degenerative diseases of CNS, were found to be associated with mortality. Stroke represented a significant risk factor for the 31-day mortality. On the contrary, HTN resulted to have a protective role in the risk of death at the 12-month FU. This paradoxical result could be explained, at least partly, by the large use of antihypertensive drugs, a treatment that efficiently lowers the risk of cerebrovascular acute events. Degenerative diseases of the CNS represent the comorbidities associated with the higher risk of death at the 24-month FU, in the univariate model, but not in the multivariate model. Only HTN, stroke, and HFRS maintained the statistical significance both in the univariate and the multivariate model. In particular, for HFRS, the “intermediate risk” category resulted in a higher mortality risk at 12- and 24-month FU, in both models (univariate and multivariate), and, unexpectedly, the high-risk category did not result associated with the mortality risk. Probably, this aspect could be dependent upon the limited number of patients belonging to this category. Regarding the analyzed microRNAs, only miR-17 and miR-126-3p showed a significant inverse association with the short- and medium-term mortality risk. High expression levels at hospital admission of miR-17 were associated with a better prognosis at 31-day and 24-month FU. The most relevant result concerns, however, miR-126-3p: high expression levels at admission were associated with a reduced mortality risk at 12 and 24 months. The significant association with mortality was confirmed by multivariate Cox Proportional hazards regression analysis, in which age, gender, NLR, eGFR, and HTN were included.

MiR-126-3p is an angiogenetic regulator abundant in endothelial cells (ECs) and endothelial progenitor cells (EPCs). miR-126 expression was identified as a strong and independent predictor for long-term all-cause mortality among patients with T2DM [36]. Interestingly, low miR-126-3p levels have been linked to CKD mortality, CVD complications, kidney disease progression, and premature death risk due to cancer and cardiovascular disease [37, 38].

Overall, our results reinforce the increasing evidence suggesting that low miR-126 circulating levels are related with endothelial dysfunction and high systemic inflammation, two key risk factors for unhealthy aging.

A deeper insight into the possible pathways targeted by miR-126-3p and miR-17 was analyzed by DIANA-miRPath v.4, an online miRNA analysis tool. Pathways targeted by miR-126-3p and miR-17 are related to distinct mechanisms associated to multimorbidity, i.e., autophagy, longevity, cancer, and cellular senescence. The analysis of the seven mRNAs targeted by the miRNAs revealed further association with age-related human diseases. For instance, loss of Dicer contributes to cardiovascular diseases and has a crucial role in RNA-based antiviral immunity [39]. E2F-1 and E2F-3 are shown to be important regulators for cell proliferation [40], particularly E2F-1, which has been demonstrated to improving myocardial hypertrophy [41].

As key regulators of apoptosis, MCL-1 and BCL-2 are survival factors and thus considered ideal cancer targets [42]. IGF2BP1 has been linked to coronary artery disease (CAD) and T2DM, and it has been proposed as a potential therapeutic target in atherosclerosis and diabetic angiopathy [43, 44]. Lastly, CRK is an adaptor protein which is involved in T-cell adhesion and migration [45].

In this framework, the evaluation of c-miRNA levels in the elderly could be of relevance to unreveal the mechanisms and processes for multimorbidity development and progression.

Interestingly, among classical blood biomarkers, eGFR and NLR represented two strong risk factors of mortality, with eGFR as the risk factor for 31-day mortality, confirming previously results [46] and NLR representing a risk factor of medium-term death. Previous reports showed that circulating biomarker levels could be negatively related to eGFR, especially in geriatric patients affected by age-related diseases and infectious [47]. However, this does not preclude the potential role of specific circulating miRNAs in the stratification of geriatric patients based on mortality risk.

Regarding NLR, this biomarker can be considered a surrogate marker for systemic inflammation, and it has recently gained increasing public interest, since it is associated with several comorbidities, including insulin resistance, cardiovascular disease (CVD), prevalence and incidence of type 2 diabetes, and CKD progression [48, 49]. NLR is a promising biomarker, easily available in clinical practice and with a high effectiveness-cost ratio and good reliability due to its lower variability by treatments [50]. Although there is no consensus of what the normal NLR levels are, some studies identified > 5 as the cut-off value to predict adverse outcomes. It was previously proposed that an NLR ≥ 5 as a global index of inflammatory-immunological status may be a useful marker for screening and preventing MM [51]. Overall, we identified circulating biomarkers, such as miR-17, 126-3p expression levels, and NLR, significantly associated with mortality in the setting of geriatric hospitalized patients.

## Limitations and conclusions

A limited number of patients, all affected by cardiovascular MM, were selected for this study. However, it is important to note that these patients are very old, with a median age of 86 years. Notably, MM still represents a challenge for the physicians, and strengthening the research for new minimally invasive biomolecular markers related to mortality risk in geriatric patients with MM is an urgent need. Future studies with larger cohort could eventually help to identify potential novel biomarkers to implement risk stratification.

### Supplementary Information

Below is the link to the electronic supplementary material.

Supplementary file1 (DOCX 16 KB)
