# Supplementary material for: Low circulating levels of miR-17 and miR-126-3p are associated with increased mortality risk in geriatric hospitalized patients affected by cardiovascular multimorbidity
Source: GeroScience. 2023 Nov 27;46(2):2531–44. doi: 10.1007/s11357-023-01010-1 (PMC10828307; doi:10.1007/s11357-023-01010-1)
Supplement: Supplementary file 1 — Supplementary file1 (DOCX 16 KB) [file 11357_2023_1010_MOESM1_ESM.docx]

Supplementary materials:

**Supplemental Table 1**. List of the 17 comorbidities and corresponding ICD-9 codes

| Condition description | ICD-9 codes |
| --- | --- |
| CHF | 402.01 402.11 402.91 404.11 404.91 428.0 428.1 428.22 428.23 428.30 428.41 428.43 428.9 429.1 429.3 |
| HTN | 401.1 401.9 402.01 402.10 402.11 402.91 403.90 404.11 404.90 404.91 |
| Chronic kidney disease | 585.1 585.2 585.3 585.4 585.5 585.6 585.9 600.01 |
| Cardiac dysrhythmias | 427.31 427.32 427.81 |
| COPD | 491.20 491.21 491.9 |
| Diabetes/Dyslipidemia | 250.00 250.01 250.02 250.40 250.6 250.60 250.62 250.72 272.0 272.2 357.2 |
| CAD/PAD | 394.1 395.2 396.2 410.31 410.71 411.89 410.91 412 413.9 414.8 414.9 416.0 416.8 424.1 427.5 429.1 429.79 433.10 440.20 440.23 441.4 441.9 442.1 453.40 |
| Acute diseases of the digestive system | 438.82 530.10 532.00 535.01 535.10 535.11 535.40 535.41 535.50 542 558.9 560.39 560.9 564.00 575.3 576.1 577.0 787.2 787.91 789.5 |
| Endocrine, nutritional and metabolic diseases | 244.0 244.3 244.9 262 263 263.0 276.1 276.51 278 |
| Pneumonia | 482.83 482.89 482.9 485 486 507.0 |
| Degenerative diseases of the CNS | 290.0 290.10 290.20 290.3 290.40 290.41 293.0 298.2 300.4 331.0 331.2 331.83 332.0 345.10 345.50 438.0 438.9 |
| Acute diseases of the urinary system | 131.02 584.5 584.9 586 590.10 590.80 591 592.0 595.0 598.01 599.0 788.0 788.20 788.29 788.5 |
| Chronic diseases of the digestive system | 553.3 562.10 562.11 562.12 562.13 569.0 569.85 571.40 571.5 571.9 572.4 574.10 574.20 574.61 574.90 579.9 |
| Cancer | 151.9 156.1 157.9 159.0 162.9 174.9 185 189.0 198.5 202.00 202.01 203.00 204.10 204.20 205.00 205.10 211.4 211.5 284.9 289.3 289.83 V1005 1006 V1052 |
| Bone & muscle diseases | 715.09 715.15 715.16 715.90 718.20 722.2 724.2 725 728.2 729.81 733.00 733.09 733.13 |
| Unspecified pleural effusion | 511.9 511.9 512.1 |
| Stroke | 434.01 435.9 436 |

CHF, Chronic Heart Failure; HTN, Hypertension; COPD, Chronic obstructive pulmonary disease; CAD/PAD, Coronary Artery Disease/ Peripheral Artery Disease; CNS, Central nervous system.
